# An Overview of Intraoperative OCT-Assisted Lamellar Corneal Transplants: A Game Changer?

**DOI:** 10.3390/diagnostics12030727

**Published:** 2022-03-17

**Authors:** Matteo Mario Carlà, Francesco Boselli, Federico Giannuzzi, Gloria Gambini, Tomaso Caporossi, Umberto De Vico, Luigi Mosca, Laura Guccione, Antonio Baldascino, Clara Rizzo, Raphael Kilian, Stanislao Rizzo

**Affiliations:** 1 Ophthalmology Department, Fondazione Policlinico Universitario A. Gemelli, IRCCS, 00168 Rome, Italy; francescoboselli@outlook.it (F.B.); federico.giannuzzi@gmail.com (F.G.); gambini.gloria@gmail.com (G.G.); tomaso.caporossi@gmail.com (T.C.); umbertodevico@gmail.com (U.D.V.); luigi.mosca@policlinicogemelli.it (L.M.); laura.guccione@policlinicogemelli.it (L.G.); antonio.baldascino@policlinicogemelli.it (A.B.); stanislao.rizzo@gmail.com (S.R.); 2 Ophthalmology Department, Catholic University “Sacro Cuore”, 00168 Rome, Italy; 3 Ophthalmology, Department of Surgical, Medical and Molecular Pathology and Critical Care Medicine, University of Pisa, 56126 Pisa, Italy; clararizzo2@gmail.com; 4 Ophthalmology Unit, University of Verona, 37134 Verona, Italy; raphaelkilian8@yahoo.it

**Keywords:** anterior segment iOCT, intraoperative optical coherence tomography, microscope-integrated optical coherence tomography, lamellar keratoplasty, DALK, DSAEK, DMEK

## Abstract

Intraoperative optical coherence tomography (iOCT) is a noninvasive imaging technique that gives real-time dynamic feedback on surgical procedures. iOCT was first employed in vitreoretinal surgery, but successively served as a guidance in several anterior segment surgical approaches: keratoplasty, implantable Collamer lens (ICL) implantation, and cataract surgery. Among all of those approaches, the unbeatable features of iOCT are fully exploited in anterior and posterior lamellar keratoplasty, and the purpose of this review is to focus on the advantages and shortfalls of iOCT in these techniques, in order to assess whether this technology could be a real step forward. In deep anterior lamellar keratoplasty (DALK), iOCT is useful to evaluate the needle depth into the corneal stroma, the big bubble dissection plane, and residual stromal bed, thus aiding the standardization of the technique and the reduction of failures. In Descemet stripping automated endothelial keratoplasty (DSAEK), iOCT allowed for clear visibility of fluid at the graft/host interface, allowing for immediate rescue maneuvers and granting the best graft apposition. In Descemet membrane endothelial keratoplasty (DMEK), iOCT can track the lenticule unfolding in real time and assess graft orientation even in severe hazy corneas, thus optimizing surgical times, as well as avoiding the use of potentially hazardous exterior markers (such as the “S” stamp) and preventing unnecessary manipulation of the graft. Overall, the role of iOCT appeared crucial in several complicated cases, overcoming the difficulties of poor visualization in a fast, non-invasive way, thus raising this approach as possible gold standard for challenging conditions. Further improvements in the technology may enable autonomous centering and tracking, overcoming the current constraint of instrument-induced shadowing.

## 1. Introduction

Because of their inherent advantages over full-thickness penetrating keratoplasty (PK), lamellar keratoplasty techniques have gained traction in the area of corneal transplantation. In particular Descemet membrane endothelial keratoplasty (DMEK) and Descemet stripping automated endothelial keratoplasty (DSAEK) have become the most popular procedures for treating corneal endothelial dysfunction [1], while in the presence of healthy endothelium, deep anterior lamellar keratoplasty (DALK) has become a preferred alternative to PK for the treatment of corneal ectasias and other stromal disorders [2]. Nevertheless, these surgeries still require an important learning curve and carry relevant postoperative complications [1,3,4].

In recent times, anterior segment optical coherence tomography (AS-OCT), allowing high-resolution, cross-sectional images of the AS, has gained an important role in the follow-up of lamellar corneal transplants, offering a clear evaluation of graft positioning and attachment [5,6,7,8]. Intraoperative OCT (iOCT) is a new method that has the potential to transform surgical approaches by delivering real-time dynamic feedback on tissue changes during operation [9,10]. While iOCT has been used extensively in posterior eye surgery, iOCT in the anterior segment of the eye is still a developing area. The newly developed microscope integrated iOCT smoothly integrates image capture while conducting surgery, allowing for real-time evaluation of different surgical processes and demonstrating a way for assessing the qualitative and quantitative impact of surgical interventions on tissues [11].

The purpose of this review is to evaluate advantages and shortfalls of iOCT for lamellar corneal transplants, in order to assess whether this technology may become a real game changer in these procedures, minimizing intra- and post-operative complications through an improved visualization of anterior segment ocular structures. We conducted a comprehensive literature search in MEDLINE, focusing on papers matching the keywords “intraoperative optical coherence tomography” or “iOCT” and “DALK”, “DSAEK”, “DMEK”, “lamellar keratoplasty” or “endothelial keratoplasty”.

## 2. iOCT in Anterior Segment Surgeries

Izatt et al. described anterior segment OCT (AS-OCT) imaging for the first time in 1994 [12], utilizing the same 830 nm wavelength of light as retinal OCT. Because this approach suffered from low penetration into scattering tissues such as the sclera, a new 1310 nm super luminescent diode approach was presented, offering increased penetration through the sclera and real-time imaging at an 8-frame-per-second rate [5]. AS-OCT offered several advantages in the diagnostic evaluation of the anterior segment: measurement of anterior chamber biometry, angle configuration, corneal thickness, and lens thickness, views of pathological processes, and examination of post-surgical anatomy and post-traumatic eyes. As a noncontact approach, its use also appeared to be feasible during surgical times.

Both anterior and posterior segment procedures initially benefited from the use of handheld OCT instruments, although this approach determined prolonged surgical times [9]. Finally, OCT devices were incorporated into surgical microscopes [13], delivering a high-quality picture in the surgeon’s oculars on demand, rather than only on a nearby display, making it easier to rely on the OCT image while working without having to take the eyes away from the surgical field [11]. Various surgical microscope integrated optical coherence tomography (Mi-OCT) devices are now commercially available [14], such as the ZEISS OPMI LUMERA 700 (Carl Zeiss Meditec, Inc., Oberkochen, Germany) or the Leica Proveo 8 (Leica Microsystems, Wetzlar, Germany), allowing for both external video display panel visualization and heads-up integrated vision into the microscope oculars.

iOCT was first employed in vitreoretinal surgery [15,16], focusing on macular hole and retinal detachment surgery [17,18]. Furthermore, iOCT has been effectively used for decision assistance in several anterior segment surgical approaches: patients undergoing keratoplasty, implantable collamer lens (ICL) implantation, and cataract surgery [19,20,21,22,23,24,25,26,27,28]. Some other applications of iOCT can be as support in assessing corneal incisions, intra-lenticular pressure, and posterior capsule integrity during phacoemulsification; creating precise partial-thickness scleral flaps; and establishing a diagnosis and performing surgery in pediatric patients who are not cooperative during examination [29]. iOCT has recently been introduced also to the area of glaucoma surgery, allowing vision of trabecular aspiration, ab interno trabeculotomy or visualizing angle structures during the placement of aqueous shunts; nevertheless, for intraoperative usage, wavelength modifications and oblique scanning are required [30].

Nevertheless, among all of those approaches, the unbeatable features of iOCT are fully exploited in anterior and posterior lamellar keratoplasty.

## 3. iOCT-Assisted DALK

Deep anterior lamellar keratoplasty (DALK) is the gold standard surgical method for corneal ectasias or stromal dystrophies, according to the American Academy of Ophthalmology, since it delivers visual benefits similar to those of penetrating keratoplasty while being safer [31,32].

The effectiveness of DALK relies on keeping the Descemet membrane (DM) intact while leaving as little stroma as possible. To accomplish maximal stromal dissection, the most performed procedure is the big bubble (BB), firstly described by Anwar and Teichmann [33], in which air is injected through a cannula tip into the corneal stroma to separate DM or DM and a layer of deep corneal stroma known as the pre-Descemetic layer (Dua’s layer, DL). This permits the damaged stroma to be replaced with healthy stroma from a cadaver donor. This procedure can determine three forms of BB: (1) type-1, where air separates the deep posterior stroma from the anterior surface of the DL, and the BB begins in the center and expands centrifugally to a maximum diameter of 8.5 mm; (2) type-2, in which air detaches stroma from DM, beginning at the corneal periphery and expanding for a maximum diameter of 10–10.5 mm throughout the cornea’s posterior surface, but usually being partially complete; (3) mixed BB, which includes both type-1 and type-2 aspects [34]. The success rate of producing a large bubble has been reported to range from 66% to 90% [33,35], with rates varying depending on disease pathology and surgeon expertise. The phase of big-bubble production is the most typical surgical stage in which DM perforation occurs, especially for rookie surgeons, highlighting the need for a more repeatable, regulated, and safer strategy [36].

iOCT stands out as a means of standardization during several steps of DALK, in order to optimize success rates and minimize PK-conversion rates [37]. Geerling et al. published the first paper documenting the use of iOCT during a DALK procedure in 2005. All phases of the surgical technique could be seen intraoperatively, although the creation of shadows under the metallic instruments utilized was an apparent constraint [6]. iOCT aided in the measurement of the initial depth of trephination and the depth of needle insertion, allowing for the development of the big bubble, but also, in the preparation on the Descemet membrane, the placement of the donor tissue and the verification of interface fluid [22,37].

**Figure 1:**
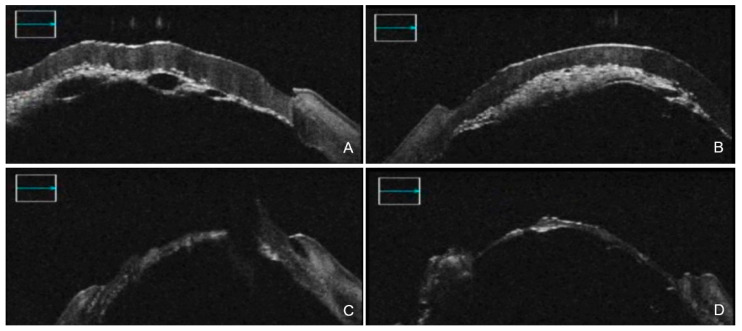
iOCT-assisted DALK conducted by an expert surgeon. After air injection (**A**), a failure in big bubble formation was visible thanks to iOCT, with different areas of residual adherence between posterior stroma and DM (**B**). iOCT helped the surgeon perform manual dissection of the planes (**C**) and final assessment of the uniformity of DM (**D**). iOCT = intra-operative optical coherence tomography; DALK = Deep anterior lamellar keratoplasty; DM = Descemet membrane.

When performing DALK in cases where a big bubble should not be attempted or cannot be achieved, iOCT is especially useful because it allows the surgeon to better control the incision depth and assess the uniformity of the dissection plane to optimize visual outcomes, especially when coaxial microscopy does not offer excellent evaluation of the depth of a corneal incision [20,23]. Furthermore, knowing which type of bubble had developed intraoperatively is critical, because the type-1 and type-2 components of a mixed bubble are prone to tearing and bursting. In this situation, iOCT can direct the surgeon to a route change, in order to find a more appropriate way to conclude the procedure [20] (Figure 1).

In 2013, Scorcia and his colleagues highlighted the results of the intraoperative AS-OCT-guided big bubble technique in 100 consecutive patients treated with DALK, focusing on several parameters: stromal depth reached with the cannula tip, success rate in achieving big bubble formation, and complication rate. The average distance between the cannula tip and the DM was found to be 104.3 ± 34.1 µm thanks to the AS-OCT during operation. The remaining tissue thickness in the group of patients who developed a big bubble (mean, 90.4 ± 27.7 µm) was substantially smaller than in the group of unsuccessful surgeries (136.7 ± 24.2 µm) [2]. Based on these findings, they suggested that the evaluation of the depth of injection may induce changes in surgical strategy, aborting pneumatic dissection if the cannula insertion site is shown to be too superficial, and instead trying a secondary insertion to reach a plane deep enough for effective big bubble generation. AS-OCT scans offer the reliability of being repeated until the cannula is positioned in the correct plane, since alignment and scanning times can be achieved in less than 30 s. Nevertheless, success rates for iOCT-guided big bubble formation were comparable to other studies without iOCT (70%), nor was the rate of macro- and micro-perforations lower than described in the literature [2].

Steven et al. successively described step-by-step monitoring of DALK procedures with iOCT: the depth of trephination could be properly observed, and needle insertion in close proximity to DM could be consistently monitored. Successively, air injection into the posterior stroma was detected and appeared as a yellowish staining of the OCT picture, a result of the enhanced tissue scattering induced by air bubbles [38]. Stromal tissue preparation for smoothed DM obtainment, either for graft insertion or suturing, could be analyzed through iOCT, with a final control on interface congruence to be conducted. In four cases, iOCT detected small air bubbles distributed over the whole corneal stroma, but no big bubble. In these cases, a 15° knife was used to incise individual air bubbles to generate a bigger cavity, and this allowed a rescue in two of the four cases, with no need for PK conversion. Moreover, in two cases, iOCT was able to detect the remaining interface fluid after graft placement, allowing the surgeon to safely evacuate the residual fluid by moderate massage of the corneal surface and blunt opening of the interface in all four quadrants, under air tamponade of the anterior chamber [22]. These findings were consistent with other studies that claimed iOCT provided an advantage in visualizing the host–donor apposition and management of any interface fluid or debris under direct observation, resulting in lowered risk of a double anterior chamber in the postoperative period, perhaps avoiding the need for a second surgical procedure.

Au et al. recently analyzed the feasibility and utility of iOCT in the DALK subgroup of the PIONEER Study. Eighteen eyes were evaluated, with four of them suffering from significant corneal scarring. Of the 18 attempted big bubbles, 11 eyes had a successful procedure, while the others underwent PK conversion. Although not every eye had imaging conducted at each time point, iOCT imaging was successfully acquired in all 18 eyes, with an average of 3.8 scans for a total extra time spent of 284 s and no iOCT-related intraoperative complications. iOCT trephination depth evaluation resulted in a decision in 40% of the cases to manually deepen the dissection prior to tunnel creation, according to surgeon feedback forms [23]. Although not statistically significant, in comparison to PKP, properly completed DALK had a 10% larger percentage trephination depth. On the other hand, deeper cannula tracts were not correlated in this study with successful DALK operations, but measurement of the cannula tip’s distance from the pre-DM plane appeared to be a helpful approach to real-time evaluation of the ideal air injection site [23]. The existence of intrastromal emphysema following air injection was shown to reduce visualization of deeper tissues, restricting input to the surgeon, similarly to the aforementioned shadowing effect of the metallic cannula [23].

iOCT was also claimed to be useful for Descemet’s membrane detachment (DMD), which is a frequent postoperative complication after DALK, especially in situations where the original operation was complicated by a micro-perforation. In fact, iOCT-assisted injection of isoexpansile gas into the anterior chamber to treat DMD has been described, aiding in the selection of the location for venting incisions to drain the interface fluid, thereby confirming the DM’s reapposition after surgery [24].

In recent years, Ehlers et al. in the DISCOVER study conducted a 3-year prospective assessment of the feasibility and utility of microscope-integrated iOCT during ophthalmic surgery, focusing on standardized surgeon feedback surveys immediately after the procedure. The needle depth into the corneal stroma, the big bubble dissection plane, and residual stromal depth in the cornea bed were the most-often reported areas of importance in DALK, with real-time OCT the preferred mode of image acquisition (78.3% of cases) rather than static iOCT images (17%) [11].

One of the most recent approaches was reported by Liu et al. and consisted of a DALK procedure through the use of low-energy femto-second laser (FSL) and iOCT to offer safe, accurate, controllable, and reproducible lamellar and tunnel cuts to a chosen depth in a single step, allowing for air injection in big-bubble DALK procedures [39]. After a pre-clinical stage in which 30 porcine eyes were used for the evaluation of the accuracy of tunnel creations, 14 human eyes were included in the clinical stage. Targeted distances of 50 and 120 µm between the tunnel end and DM (D_t−dm_) and between the tunnel end and lamellar cut (D_t−l_) were set respectively, relying on iOCT evaluation, after confirming good accuracy of laser settings in porcine eyes (<10% deviation from the targeted D_t−dm_). In this study, this technique allowed the surgeon to achieve 100% type-I big bubble formation [39]. Moreover, higher accuracy of intrastromal tunnel depths, thanks to the real-time in-built OCT, was observed when compared to Scheimpflug imaging-aided FSL-DALK presented in another study [40].

Ultimately, Santorum et al. conducted an interventional case series in 16 consecutive eyes of 16 patients with keratoconus, using iOCT for real-time quantitative analysis of surgical planes in the BB approach, aiding trephination groove extension to a mean depth of 118.9 ± 27.1 μm from the endothelial surface, compared to an average of 257.1 ± 42.5 μm before iOCT utilization [41]. This allowed for a 75% rate of success in BB formation, reaffirming the role of iOCT in deciding whether to use pneumatic dissection or expand the trephination groove, and allowing the injection cannula to be inserted at the appropriate stromal depth [41].

## 4. iOCT-Assisted Endothelial Keratoplasty

iOCT serves as a guidance tool for key surgical processes in endothelial keratoplasty, from scoring the Descemet membrane to guaranteeing graft apposition at the conclusion of the procedure. For this reason, both Descemet stripping anterior endothelial keratoplasty (DSAEK) and Descemet membrane endothelial keratoplasty (DMEK) are made easier by intraoperative OCT [21,27,42,43].

The generation of thin lenticules to improve visual results following DSAEK derives from a double-pass approach, proposed 10 years ago, that entails cutting the donor tissue twice with various microkeratome blades [44]. Following the initial microkeratome pass, the surgeon can use iOCT to examine the residual donor thickness and choose the best blade size for the second microkeratome pass. This aided in the creation of ultra-thin donor lenticules while reducing the risk of donor tissue perforation. Even when a single microkeratome pass is utilized to prepare the donor tissue, iOCT reliably measures the donor thickness and aids in the selection of the best blade size [26].

Moreover, different methods of improving Descemet membrane (DM) visualization have been documented, including the use of trypan blue dye to stain the DM and the use of a crescent blade as a reflector [45]. In situations of highly edematous corneas, iOCT assisted in the detection and elimination of DM. In circumstances when the DM is fibrosed and scarred, remnant DM tags can be easily detected and removed under direct sight of iOCT images.

During graft implantation, iOCT can track the lenticule unfolding in real time. This is especially beneficial in extremely edematous corneas with inadequate sight, when the graft might open in an inverted orientation, resulting in surgical failure. Although tiny donor lenticules are associated with better visual results, managing them is more challenging, and thin lenticules have a tendency to fold and roll. The thorough apposition of the donor lenticule is a vital step in determining the outcome of surgery, and numerous methods have been documented to achieve total removal of interface fluid, including corneal massage, internal air tamponade, and venting incisions, all of them clearly documented with iOCT [10,25,28,46]. Furthermore, in situations of bullous keratopathy, an iOCT can be used to determine the real stromal thickness, as the hypertrophic epithelium produces a falsely high total corneal thickness with conventional investigative modalities [29].

### 4.1. iOCT-Aided DSAEK

The first results of handheld intraoperative AS-OCT during DSAEK surgery were reported in 2010, when Knecht et al. highlighted the role of this technique to detect that there was still separation between the donor and host cornea at the conclusion of the procedure in two patients out of six, with an average width of interface fluid of 40 µm, despite following several published guidelines for good graft adhesion during surgery [8]. Other case studies have employed iOCT to demonstrate interface fluid outflow after corneal stab wounds [47,48], with Ide et al. focusing on the study of intraoperative fluid dynamics [7].

The PIONEER study was fundamental to highlight the utility of a microscope-mounted iOCT in different fields of ophthalmic surgery [9]. In DSAEK, iOCT allowed for clear visibility of fluid at the graft/host interface, in order to grant the best graft apposition, with the operation to be prolonged until optimum fluid evacuation was attained. On the first postoperative day, 3% (5/143) of the grafts dislocated. In all cases, a surgeon feedback form was completed and resulted in a 64% rate of uncertainty of graft apposition from the surgeon. Thanks to iOCT, persistent fluid was found in 48% of those cases, necessitating repeated procedures. Overall, in 65/135 (48%) eyes, iOCT determined surgical strategy changes, due to the evidence of persisting interface fluid necessitating further interventions, which was reported also in 29% of cases in which the surgeon believed the graft was completely apposed [9]. Hallahan et al. exploited the same PIONEER study cohort to analyze intraoperative interface fluid dynamics and correlate fluid parameters with graft non-adherence during the first week. iOCT measurements showed that total fluid volume was significantly higher when comparing completely or partially non-adherent grafts (0.22 and 0.17 µm^2^) with grafts that had no post-operative interface fluid (0.05 µm^2^). Similarly, the maximum area of fluid in the final iOCT also differed with the same trends (0.07 and 0.06 µm^2^ in eyes with subsequent displaced grafts and partial nonadherence grafts vs. 0.03 µm^2^ in eyes with totally adhered grafts) [49]. Despite the fact that there were no significant variations in results between the full and partial dislocation groups, the complete dislocation group tended to have higher interface fluid levels. Within the first week after surgery, many iOCT parameters were linked to whole and partial graft nonadherence, such as increased final interface fluid volume and area, maximum isolated interface fluid pocket volume, and mean and maximum interface fluid thickness, with volumetric and area assessments of final interface fluid more strongly associated with adherence graft rates rather than linear parameters. At last, there were no interface fluid characteristics linked to graft rebubbling and regrafting [49].

Successive studies were reported with the use of Mi-OCT, which offers better image stabilization compared to microscope-mounted or handheld iOCT in previous studies. Steverink et al. reported a case series of 8 eyes undergoing Mi-OCT-assisted DSAEK, which allowed the surgeon to identify persisting fluid interface in six cases, successfully managed with corneal swiping with a subsequent decrease in maximal interface width [50].

**Figure 2:**
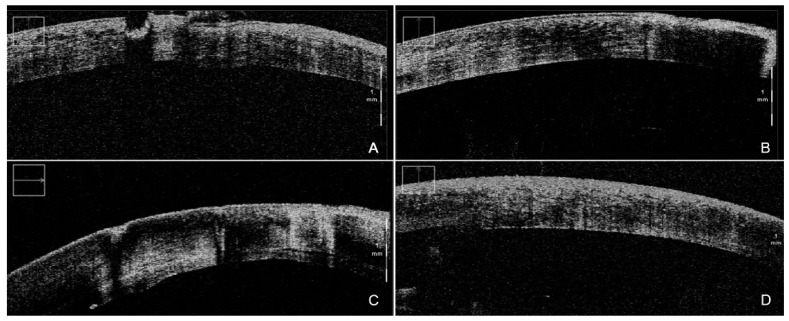
iOCT-assisted DSAEK performed by an expert surgeon. During DM stripping, iOCT aided debris visualization (**A**) in order to obtain the smoothest possible dissection plane (**B**). After the implantation of the graft, iOCT was able to identify even the smallest persisting interface fluid (**C**), thus allowing for complete apposition of the graft, clearly visible at the end of the surgery (**D**). iOCT = intra-operative optical coherence tomography; DSAEK = Descemet stripping automated endothelial keratoplasty; DM = Descemet membrane.

The most important research regarding microscope-integrated iOCT led to the publication of the DISCOVER study. In this research, Mi-OCT was reported to provide valuable feedback in 88.5% of cases, with interface fluid extent evaluation the most-cited area of iOCT feedback. The surgeon, by means of standardized questionnaires, declared the graft to be clinically apposed in 68.3% of patients in the DSAEK subgroup. In 46 cases (54.8%), however, residual interface fluid was seen on iOCT, thus permitting rescue procedures [11]. Further analyses were conducted with the same cohort of the DISCOVER study, with Ehlers et al. reporting that Mi-OCT aided decision-making during DSAEK surgery in 41% of cases, in which further maneuvers were necessary based on iOCT. When the surgeon thought the graft was entirely apposed clinically, iOCT revealed residual fluid in 19% of instances. Conversely, iOCT indicated full apposition in 47% of instances where the surgeon did not believe the graft was completely apposed, saving time and needless adjustments during surgery [10] (Figure 2).

A recent retrospective study by Asif et al. focused on Mi-OCT-assisted DSAEK in congenital hereditary endothelial dystrophy (CHED) [51]. Corneal transplantation in infants may be difficult owing to a variety of ocular characteristics, including a tiny eyeball, a very low anterior chamber, enhanced positive posterior pressure, poor scleral stiffness, phakic status, and limited intraocular space. These variables may increase the likelihood of problems [52]. Moreover, CHED patients often have poor visibility due to corneal edema and stronger adherence of the DM to the underlying stroma, in contrast to decompensated corneas of Fuchs’ endothelial dystrophy [53]. This might cause DM residues to remain in the graft, obstructing graft apposition. iOCT proved useful in these conditions, thereby improving the outcomes of the procedure. Among the 39 eyes undergoing DSAEK, the graft was attached intraoperatively in all cases, while graft detachment was seen in the early postoperative phase in 4 eyes, requiring a rebubbling procedure [51].

### 4.2. iOCT-Aided DMEK

For the treatment of corneal endothelial disorders, Descemet membrane endothelial keratoplasty (DMEK) outperforms Descemet stripping automated endothelial keratoplasty (DSAEK). DMEK, in particular, has been demonstrated to speed up visual rehabilitation, improve visual acuity, and reduce corneal rejection [54,55,56,57]. Despite its benefits, DMEK necessitates the acquisition of new skills by the surgeon, which may result in a high learning curve [55,58]. DMEK scroll orientation may be seen and identified in real time using iOCT, avoiding the use of potentially hazardous exterior markers (such as the “S” stamp or peripheral notches), and may be useful in cases of corneal opacity or advanced corneal edema when vision is impaired [21,27,59].

Saad et al., in 2015, were the first to describe iOCT-assisted DMEK in a prospective case series of 14 eyes. Using the iOCT integrated into a surgical microscope (RESCAN 700; Carl Zeiss Meditec), which offered an 840-nm central wavelength that performed 27,000 A scans per second, graft orientation was correctly evaluated in all cases. The success rate was 100%, with only one patient needing rebubbling at 1 week. The mean unfolding time calculated from the moment the graft was inserted into the anterior chamber to the time the air bubble was injected into the anterior chamber, including the time spent looking at and evaluating the OCT pictures, was reported to be 6.1 min, faster when compared to the 8.9 min needed by the same surgeon without this technology [27]. Even in difficult situations with severe corneal edema, when direct visibility of the graft was hindered, OCT pictures aided graft orientation assessment, suggesting that live OCT aid may also assist in broadening DMEK indications in severe corneal edemas. Moreover, in the instance where the graft was in the incorrect orientation, OCT pictures enabled the surgeon to confirm graft rotation [27].

The aforementioned DISCOVER study evaluated the role of iOCT even in DMEK procedures. In the report on the first eight eyes, iOCT-assisted DMEK allowed completion of intraoperative graft attachments in seven eyes, while one graft exhibited a linear region of non-adherence matching a large posterior stromal irregularity, clearly visible on iOCT during descemetorhexis. The median “unscrolling time” was 6 min and 15 s, and the graft orientation was rapidly identifiable in 100% of cases [59].

These results were confirmed at 3-year follow-up in the DISCOVER study, in which surgeons reported iOCT-enabled graft orientation feedback in 63% of instances in DMEK, based on the scrolling arrangement of the tissue. Moreover, iOCT was commonly used to establish orientation even before viewing of the orienting “S” stamp, thus allowing surgeons to abandon the use of this stamp [11]. In addition, following first implantation, the surgeons reported clinical graft apposition in 54 patients (90%). Intraoperative imaging revealed persisting interface fluid in 7% of cases, allowing for further corneal sweeping before the surgery was completed. In this research, the trend of preferred iOCT visualization was analyzed: surgeons used the external screen in 30% of cases in the first year, the heads-up display in the oculars in 25% of cases, and both kinds of vision in 42% of instances. In years 2–3, a substantial change toward using the screen occurred in 82% of instances, compared to oculars in 10% and both systems in 8% [11].

In the last update of the DISCOVER study, those findings were confirmed. Patel et al. noted that iOCT offered helpful real-time feedback in all instances (100%) and did not interfere with the surgical operation in any manner [43]. In all instances, the iOCT picture on the linked external video display was preferred, because of the smaller, lower-definition picture on the inside display. iOCT also confirmed its effectiveness by allowing for quick and safe detection of tissue orientation in 99% of cases, thus avoiding other reported marking techniques [60,61]. Moreover, average unscrolling time of 4.4 ± 4.1 min was competitive when compared to reported times for S-stamped grafts (5.7 ± 3.9 min) and for non-stamped grafts (6.4 ± 4.4 min) [59,62]. The rebubbling rate reported in this study was 6.4%, suggesting that iOCT-assisted DMEK may yield equivalent or better early outcomes than previous reported outcomes, with a mean rebubbling rate of 28.8% reported in a recent thorough literature analysis across 17 DMEK case series without iOCT support [63].

iOCT favorability was also claimed by Muijzer et al., who reported recent experience with 22 iOCT-aided DMEKs with a reduced over-pressurization time (<2 min, compared to the >12 min of the standard procedure) [64]. Thanks to the capacity of iOCT in reducing unscrolling times, as reported by previous studies, they decided to avoid over-pressurizing the globe for an extended duration during surgery, since a high intra-ocular pressure (IOP) may harm corneal endothelial cells and the retinal nerve fiber layer [65,66]. Refraining from over-pressurization enabled reducing the entire surgical time by 24% without endangering its safety or compromising clinical outcomes. Moreover, iOCT provided the surgeon with useful information, including an unclear graft orientation in 12 cases and interface fluid and minor graft detachments in 8 instances. Ultimately, iOCT altered the surgical decision-making process in 42% of cases, and its role in the management of graft misorientation or in the prevention of unnecessary manipulation was crucial [64].

A recent prospective study was conducted by Sharma et al., specifically targeting 25 cases of corneal decompensation with poor visualization due to pseudophakic bullous keratopathy, Fuchs endothelial corneal dystrophy, failed graft or iridocorneal endothelial (ICE) syndrome that underwent Mi-OCT-guided DMEK [42]. Prior to descemetorhexis, regions of lacking DM in the host cornea were identified thanks to Mi-OCT, preventing needless DM scraping, which might have resulted in postoperative stromal haze. After completing the descemetorhexis, retained DM tags, otherwise undetectable, were detected on Mi-OCT in 92% of instances (23 eyes) and immediately removed using micro-vitreoretinal forceps and a vitrectomy cutter in all of these patients. In the case of ICE syndrome, Mi-OCT guided evaluation and release of peripheral and mid-peripheral anterior synechiae. In this research, Mi-OCT was able to identify the position and form of the DM roll in the cartridge in every case, with the majority of the instances having a double scroll (76%), and minor percentages of tight scroll and trifold. Following graft implantation, the correct orientation was always detectable, even in cases of severe stromal haze, thus overcoming the shortfalls of the numerous techniques previously proposed in cases of poor visualization, such as chandelier or trans-corneal illumination [67,68,69]. iOCT proved useful to assess whether or not to try to unfold the peripheral DM folds based on their precise position and extent, avoiding inadvertent damage to the graft. This study reported graft attachment success rates of 72% at day 3, with 16% of eyes requiring rebubbling, leading to a 100% rate of attachment at the 6-month follow-up, thus underlining how Mi-OCT assistance allowed for the safe and effective conduct of the procedure, otherwise rendered unsuitable for DMEK due to the presence of severe corneal edema and poor visibility of the anterior segment details [42].

## 5. Limitations, Strengths and Future Prospects

The biggest limitation to the wider use of iOCT is its pricing and limited availability [11,29]. Because of technological developments, handheld and microscope-mounted OCT devices have been replaced by microscope-integrated iOCT, which completely combines image capture with the scanning process. Even if microscope integration allowed for additional significant advancement in the technique, such as real-time imaging surgical maneuvers with immediate feedback to the surgeon regarding instrument localization, further technology improvements may enable autonomous centering and tracking. El-Haddad et al. created a prototype automated stereovision surgical instrument tracking system, which automatically focuses the iOCT scan field on the surgical tool tip and enables continuous observation of instrument–tissue interactions across a 2500 mm^2^ field [70]. Another shortfall of iOCT technology is the current constraint of instrument-induced shadowing, which makes it difficult to see structures underlying the metallic tools, restricting the utility of iOCT during intrastromal needle insertion or lamellar dissection [9,29]. With new technology advancements and the use of plastic cannulas that avoid shadowing, these limitations may indeed be solved in the future [2].

Furthermore, there is a lack of large randomized controlled trials demonstrating the superiority of microscope-integrated iOCT over microscopy alone [71]. Despite this, our research highlighted that authors appreciate how iOCT is able to standardize surgical phases in these procedures, offering new management and optimization possibilities, as well as more data regarding depth of trephination, possible flap dislocation and fluid persistence among layers. The role of iOCT appeared indeed crucial in several complicated cases, overcoming the difficulties of poor visualization in a fast, non-invasive way, thus highlighting this approach as a possible gold standard for challenging conditions.

## 6. Conclusions

Several studies evidenced that intraoperative OCT was a big step forward in both anterior and posterior segment surgery. As regards lamellar corneal transplants, iOCT was reported to be feasible and useful in surgical decision-making, offering much more information rather than simple clinical examination, especially for challenging conditions. Microscope-integrated OCT is more accurate than operator perception in detecting minor anatomic characteristics that may influence surgery success rates and possible post-operative complications. Furthermore, iOCT technology may help to better understand and customize critical aspects of these surgical procedures (i.e., DALK big bubble injection plane) and improve clinical outcomes, thus enabling a significant step forward in the long run to the establishment of iOCT as a standard in ophthalmic surgery.

Nevertheless, due to the heterogeneity of the published studies (case series with small sample sizes, retrospective design, no baseline data, missing statistical data, inhomogeneous cohorts, lack of a control arm), the current level of evidence is not yet sufficient to be able to recommend the use of iOCT in every situation, until several randomized clinical trials are published.

## Data Availability

The data that support the findings of this study are available from the corresponding author, MMC, upon reasonable request.
